# Actigraphic Sleep and Dietary Macronutrient Intake in Children Aged 6–9 Years Old: A Pilot Study

**DOI:** 10.3390/nu11112568

**Published:** 2019-10-24

**Authors:** Silvia Coronado Ferrer, Isabel Peraita-Costa, Agustín Llopis-Morales, Yolanda Picó, José Miguel Soriano, F. Javier Nieto, Agustín Llopis-González, María Morales-Suarez-Varela

**Affiliations:** 1 Unit of Preventive Medicine and Public Health, Department of Preventive Medicine and Public Health, Food Sciences, Toxicology and Forensic Medicine, University of Valencia, Avda. Vicente Andrés Estellés s/n, 46100 Burjassot, Valencia, Spain; scoronadoferrer@gmail.com (S.C.F.); ivperaitacosta@hotmail.es (I.P.-C.); agustinllopis@gmail.com (A.L.-M.); agustin.llopis@uv.es (A.L.-G.); 2 CIBER in Epidemiology and Public Health (CIBERESP), Institute of Health Carlos III, Avda. Monforte de Lemos 3-5, Pabellón 11, Planta 0, 28029 Madrid, Spain; yolanda.pico@uv.es; 3 Environmental and Food Safety Research Group of the University of Valencia (SAMA-UV), Desertification Research Centre CIDE (CSIC-UV-GV), Moncada-Naquera Road km 4.5, 46113 Moncada, Valencia, Spain; 4 Unit of Nutrition and Bromatology, Department of Preventive Medicine and Public Health, Food Sciences, Toxicology and Forensic Medicine, University of Valencia, Avda. Vicente Andrés Estellés s/n, 46100 Burjassot, Valencia, Spain; jose.soriano@uv.es; 5 College of Public Health and Health Sciences, Oregon State University, 123 Women’s Building, Corvallis, OR 97331, USA; javier.nieto@oregonstate.edu

**Keywords:** dietary intake, energy, fat, protein, carbohydrate, sleep duration

## Abstract

The objective of this study was to examine the relationship between different sleep parameters and energy and macronutrient intake in school-aged children. A total of 203 children 6 to 9 years of age participated in this cross-sectional study. Anthropometric measurements were taken first. Diet was assessed with 3-day food logs and sleep was measured with a questionnaire on sleep quality and a wrist actigraph worn for at least 7 days. A decrease of 165.45 kcal was observed per each additional hour of sleep during the week (β (95% CI) = −165.45 (−274.01, −56.88); *p* = 0.003). This relationship was also observed for fat (β (95% CI) = −11.14 (−18.44, −3.84); *p* = 0.003) and protein (β (95% CI) = −13.27 (−22.52, −4.02); *p* = 0.005). An increase in weekend sleep efficiencies for those under the recommended threshold of 85% also had a similar association with energy (β (95% CI) = −847.43 (−1566.77, 128.09); *p* = 0.021) and carbohydrate (β (95% CI) = −83.96 (−161.76, −6.15); *p* = 0.035)) intake. An increase in habitual sleep variability was related with a slight increase in protein intake (β (95% CI) = 0.32 (0.031, 0.62); *p* = 0.031). Children who slept less had a higher energy intake, especially from fat and protein and those who presented inefficient sleep had a higher carbohydrate intake. Strategies to enhance sleep quality and quantity combined with dietary recommendations could help to improve energy and macronutrient intake levels in children.

## 1. Introduction

Adequate nutrition during childhood is es−sential to promote correct growth and development. It is vital that children are provided with a diet containing adequate quantities of energy, macro- and micronutrients to allow them reach their growth and development potential. Diet is one of the numerous factors that have been associated with childhood weight status [1,2], which, when grouped together, are known as the obesogenic environment. Another obesogenic factor that has been associated to the increase of child overweight and obesity is sleep [3,4].

Studies have observed that sleep deprivation might be one of the modifiable factors that promote inadequate nutritional intake and contribute to unhealthy food consumption behavior and excess weight gain [4,5,6]. In the last decade, the increased prevalence of obesity has been associated with sleep curtailment [7]. Previous cross-sectional and prospective studies have identified a relationship between shorter sleep duration and increased obesity in children, even after covariate adjustment including important obesity-related behaviors [8,9]. A recent meta-analysis [10] identified that reduced sleep was consistently related to a greater risk of being overweight or obese and its effect was even more pronounced in children. In addition, recent studies have shown that not only sleep duration contributes to obesity, but also timing, quality and sleep disturbances could play an important role [11,12,13]. 

Numerous findings suggested that reduced and poor sleep were linked to increased snacking and preference for poorer food choices, including energy-dense foods and snacks with higher fat and refined sugar content [14,15,16]. With respect to macronutrient composition, sleeping less is associated with an intake of a higher proportion of calories from fats [17], however, the mechanisms underlying this relationship remain unclear. 

Some hypotheses have proposed that the relationship between reduced sleep and excess food intake is based on social, behavioral and hedonic reasons [18,19]. In an environment that offers free access to ultra-palatable foods or caloric snacks, delayed bedtime or reduced sleep increases the potential time available to eat while engaging in sedentary activities [20]. On the other hand, delayed bedtime favors later wake up times and sleep disturbances induce daytime sleepiness and tiredness, which stimulates food intake [21]. These factors influence the decrease of physical activity during the day and therefore, energy expenditure is reduced. In summary, short sleep duration would favor excess food intake by reducing opportunities for physical activity and enhancing opportunities to overeat. 

Given the possibility that children may be susceptible to sleep alterations that may favor the risk of excess food intake and that sleep is a modifiable factor, it is important to continue with research that aims to explain the influence and determine the importance of adequate sleep so that policies and interventions for nutrition management programs can be developed. Previous studies have demonstrated that sleep interventions are effective for child obesity prevention [22,23]. Accelerometers have been used in sleep research for over 20 years [24] and have been validated for use in pediatric populations [25,26,27,28]. Actigraphy allows for a more objective recording of sleep parameters, such as sleep duration, efficiency, and variability, than traditional parent-report and is less intrusive than polysomnography [27]. 

The purpose of the current study was to analyze the relationship between objectively measured sleep duration, efficiency and variability in sleep duration with energy and macronutrient intake in children between 6 and 9 years old.

## 2. Materials and Methods

### 2.1. Study Design and Population

**Figure 1:**
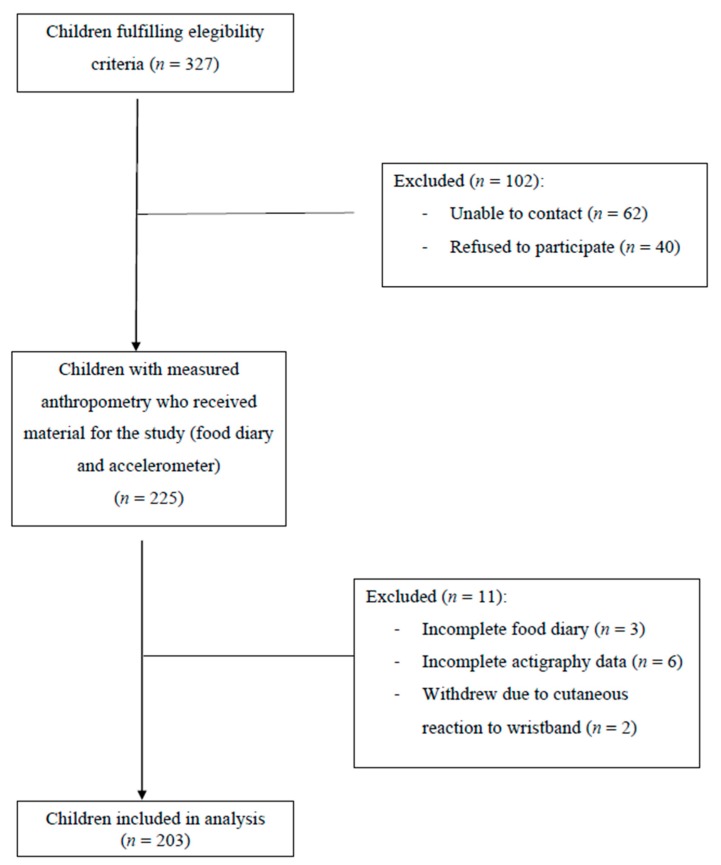
Flow diagram.

The subjects of this pilot cross-sectional study were healthy children aged 6–9 years recruited from four Spanish populations in the rural area of the province of Valencia (Figure 1). The study was approved by the Autonomous Secretariat of Education, Ministry of Education, Culture and Sports as well as the Human Research Ethics Committee of the Ethics Committee in Experimental Research of the University of Valencia (2014/29630) and respected of the fundamental principles established in the World Medical Association Declaration of Helsinki. Participants and parents or guardians were properly informed of the objectives and methods of the study and signed written informed consent prior to enrollment.

Between November 2018 and March 2019, families were invited to participate in the study by local pediatricians of the Primary Care Centers in Catadau and Alginet (Valencia, Spain). Pediatricians first contacted each family within the catchment of the primary care center with children within the eligible age range by telephone. The study was offered to the healthy pediatric population within the catchment area, children with a diagnosis of chronic illness, including sleep and breathing disorders, were excluded. Families were personally informed of the study, asked to participate, and informed consent was obtained. Then, anthropometric measurements were first collected and participants were provided with a 3-day food log, sleep log and a wrist actigraph, which was worn for at least 7 days. 

A trained pediatrician measured anthropometric variables; children were measured wearing light clothes and without shoes. Weight was measured on a digital scale (Tanita BC-545N; Arlington Heights, IL) to the nearest 0.1 kg. Fat mass was also estimated on the same digital scale by a bio impedance analysis system. Height was measured to the nearest millimeter with a wall mounted stadiometer, with children standing straight against the wall and chin parallel to the floor. Based on age, sex, weight and height, body mass index (BMI), z-score, and percentiles were calculated with WHO AnthroPlus software version 3.3.2 (World Health Organization, Geneva, Switzerland). Children were classified into 5 categories regarding BMI z-score: BMI Z-score cut-points of <−2.0, >1.0, >2.0, >3.0 are recommended to define wasted, overweight, obese and major obese [29,30]. 

Waist circumference (WC) and hip circumference (HC) were measured with a flexible but non-stretchable tape, measuring WC at the midpoint between the lower margin of the least palpable rib and the top of the iliac crest and HC around the widest portion of the buttocks. These variables were used to calculate waist to hip ratio (WHR) (WC in cm divided by HC in cm). A set of skinfold thicknesses were measured three consecutive times on the left side of the body with a skinfold caliper (Holtain Ltd.; Crymych, UK) to the nearest 0.2 mm at a constant pressure of 10 g/mm^2^.

### 2.2. Sleep Assessment

Sleep timing, patterns and duration were assessed using a questionnaire/sleep diary and actigraphy. Participants completed a parent-reported questionnaire/sleep diary that assessed sleep quality and quantity. The diary included information about wake up time, bedtime, sleep duration and sleep disturbances. Objective sleep parameters (sleep duration, efficiency and variability) were assessed using actigraphy. Children were asked to wear a GeneActiv tri-axis actigraph (Activinsights LTD, Cambs, UK) [31] on the wrist of their non-dominant hand. The GeneActiv accelerometer continuously records activity, environmental temperature and light exposure. Specific algorithm details are not available due to this being proprietary software of Activinsights LTD. The accelerometer could not be operated by the participants, nor did they have access to the data collected. The device allows for raw data to be transferred and saved as an open source or csv. The data can then be analyzed in statistical packages such as SPSS (IBM Corp., Armonk, United States) and R (R Foundation for Statistical Computing, Vienna, Austria) 

All the participants wore the actigraph continuously for 7 consecutive days and nights, and it was only removed during water or high contact sports (e.g., swimming, karate, football) which was recorded in the sleep diary. Actigraphy data was analyzed using GeneActiv Software v 1.2 (Activinsights Ltd., Kimbolton, UK) and summarized into 60 s epochs. Raw data output included acceleration in 3 axes, physical activity intensity and sleep/wake measurements. Actigraphy allows the estimation of periods of sleep measuring the lack of movement, stablished by a threshold, but it is not possible to separate the different stages of sleep. The GeneActiv actigraph has not been validated for children sleep research but its validity has been demonstrated for adult sleep studies [32,33] and also for examining physical activity in pediatric ages [34,35]. In addition, our study did not aim to study sleep problems, but to analyze the quality and quantity of sleep with dietary intake.

Recordings must have had at least 7 nights of valid actigraphic measured sleep to be eligible for analysis, including 5 weekday nights and one weekend (2 nights). Variables of interest were mean scores of sleep duration between weekdays (Sunday–Thursday) and weekend days (Friday and Saturday), calculated by actigraphy from estimated bedtime and wake-up times based on recorded movements. Another variable of interest was sleep efficiency or the time spent asleep while in bed, which depends on sleep fragmentation and sleep onset latency, compared with total time in bed, estimated by recorded movements during the time in bed period. A normal sleep efficiency is considered to be 85% or higher [36]. The last variable analyzed was habitual sleep variability, the intrasubject standard deviation of the average sleep duration across seven nights. The recommendations of sleep for the age range studied is between 9 and 12 hours of sleep daily [37,38,39]; however, a fundamental error in relating the actigraphy data sleep duration to sleep recommended hours is that recommendations are built on consensus opinion from papers that are primarily from parent report. In this study, 9 hours was taken as the recommendation following recent data on normal values for pediatric nighttime sleep measured by actigraphy for the age range studied [40]. Taking this into account, sleep duration was divided into two categories: compliant and non-compliant with sleep recommendations (≥9 h and <9 h). After, the non-compliant group was subdivided into three categories <7 h, ≥7 to <8 h, ≥8 to <9 h.

### 2.3. Dietary Intake

Families were asked to record food and beverages consumed in a 3-day food-dairy consisting of 2 weekdays and 1 weekend/holiday day. Participants were given verbal and written instructions to specify the time when food was consumed (breakfast, brunch, lunch, afternoon snack, dinner or extra snack) and the location, the type of food with brand name if possible and portion size. Dietary data was managed with DIAL software for assessing diets and food calculations for Windows, version 3.0.0.12 (Department of Nutrition UCM & Alce Ingenieria S.L.; Madrid, Spain). This validated computer tool transforms food intake data into energy and nutrients in units (g, mg, mcg)/day [41]. The main variables of interest were daily mean energy intake (kcal), carbohydrates (g), fat (g) and proteins (g). 

### 2.4. Statistical Analysis

Summary statistics of demographic characteristics were calculated as means and standard deviations. The normality of the quantitative variables was assessed using the Kolmogorov–Smirnov test. Sleep parameters (subjective duration, variability and efficiency) and dietary intake outcome variables (energy, carbohydrate, fat and protein intakes) were expressed continuously. Actigraphic sleep duration was first analyzed continuously and then by the categories it was divided into, (≥9 h and <9 h, with the latter further subdivided into <7 h, ≥7 to <8 h and ≥8 to <9 h). Available weekday and weekend data from actigraphic records were analyzed separately. Participants with implausible sleep and energy intake data were excluded from their respective analyses. 

First, the relationship between actigraphy sleep parameters, including continuous and categorical variables, and dietary intake (total energy intake (kcal/day), total carbohydrate (g/day), total fat (g/day) and total protein intakes (g/day)) was calculated as means and standard deviation. Furthermore, the relationship between actigraphic sleep parameters, subjective sleep duration and dietary intake was assessed using a multivariable linear regression analysis. The results were presented as unstandardized β-coefficients (β) and 95% confidence intervals, *p*-values <0.1 was considered statistically significant. An adjusted multivariable linear regression analysis was also carried out with sex, age, percentage of body fat and waist to hip ratio as the confounding factors adjusted for.

Since this was a pilot study, not all variables related to sleep were assessed, as focus was exclusively on dietary intake variables. In the future, anthropometric variables will also be evaluated in relation to sleep parameters in order to determine the relationship between weight and sleep. For the same reason, given the preliminary nature of the study and the small sample size, no power analysis was conducted. In the future, with a broader study of prevalence, all relevant analyses will be carried out. All statistical analyses were performed using IBM SPSS 22 Statistics for Windows (IBM Corp, Armonk, NY, USA).

## 3. Results

The general characteristics of the study population (*n* = 203), including sleep duration and nutrition data, are presented in Table 1. The mean age of the total study sample was 7.4 (Standard deviation (SD) = 1.1) years old, with girls representing 49.8% of the sample and being significantly older. The average BMI z-score of the sample was 0.8 (SD = 1.3). According to WHO criteria, 56.7% were normal weight, 25.1% were overweight and 17.8% were obese (obese: 14.8% and major obese: 3.0%). The BMI z-score did not differ significantly between the sexes while girls had a significantly higher body fat percentage than boys, and boys had higher WHR.

Regarding sleep duration, no statistically significant difference was found between the sexes. The mean weekday sleep duration was 7 h 44 min (SD = 54.6 min) and the mean duration on weekends was 7 h 44 min (SD = 1 h 5.8 min), measured by 7-day actigraphy. No statistical differences were observed when sleep duration was categorized. The mean weekday sleep efficiency was 80.5s% (SD = 8.3) and the weekend sleep efficiency was 79.4% (SD = 9.4), but statistical differences between sexes were only found in the weekday variable. The mean habitual sleep variability was 55.8 (SD = 29.2) min. 

Average daily energy consumption was 2396 (SD = 712) kcal, with macronutrients distributed as follows: protein 109.4 (SD = 60.7) g/day, fat 108.8 (SD = 48) g/day and carbohydrates 234.2 (SD = 41.5) g/day. All values strikingly exceed the daily nutritional recommendations according to the Institute of Medicine of the National Academies [42]. No statistic differences between boys and girls were observed. 

Associations between actigraphic sleep duration, efficiency and habitual sleep variability with dietary intake are presented in Table 2. The results show that those with shorter times of sleep are more likely to have a higher food consumption. When sleep duration was considered a continuous variable, significant differences were observed for weekday values of energy, fat and protein (Energy: 2397.7 (SD = 714.2) kcal, Fat 109.01 (SD = 48.04) g/day and Protein 109.5 (SD = 60.9) g/day). When sleep duration was categorized, significant differences were also observed for energy, fat and protein (Energy: 2412.6 (SD = 730.5) kcal, Fat 110.06 (SD = 49.13) g/day and Protein 110.6 (SD = 62.2) g/day). In children with a very short sleep duration (<7 h) during weekdays, mean energy, fat and protein intake were higher than in longer sleepers (Energy: 2619.1 (SD = 1328.3) kcal, Fat 122.78 (SD = 94.11) g/day, Protein 127.2 (SD = 128.4) g/day and Carbohydrates 240.97 (SD = 37.28) g/day). Similar results were observed during weekend (Energy: 2642.9 (SD = 1180.8) kcal, Fat 126.32 (SD = 82.79) g/day, Protein 123.9 (SD = 115.2) g/day) and Carbohydrates 242.62 (SD = 38.93) g/day) but without statistical significance. When weekend sleep efficiency was categorized, significant differences were also observed for energy and carbohydrates (Energy: 2364.3 (SD = 396.8) kcal, and Carbohydrates 237.4 (SD = 42.9) g/day). In this case, those with low weekend sleep efficiency consume less calories than the mean but still consume more carbohydrates. The only significant difference in relation to habitual sleep variability appears in the amount of protein intake (Protein 109.6 (SD = 61.0) g/day).

The relationship between actigraphic sleep duration, sleep efficiency and habitual sleep variability with dietary intake is presented in Table 3. The results were statistically significant for weekday parameters. A negative correlation was evident between weekday sleep duration and macronutrient intake. More precisely, for the sample as a whole and considering sleep duration as a continuous variable, a significant decrease in energy (β (95% CI) = −170.17 (−276.59, −63.76); *p* = 0.002), fat (β (95% CI) = −11.43 (−18.59, −4.20); *p* = 0.002) and protein (β (95% CI) = −13.84 (−22.90, −4.75); *p* = 0.003) intake was observed per each additional hour of sleep during the week. For the category that included all children who slept less than 9 hours a day, a significant decrease in energy (β (95% CI) = −172.76 (−293.62, −51.90); *p* = 0.001), fat (β (95% CI) = −11.53 (−19.66, −3.39); *p* = 0.006) and protein (β (95% CI) = −14.28 (−24.59, −3.97); *p* = 0.007) intake was observed per each additional hour of sleep. In children who slept less than 7 hours a day, a significant decrease in energy intake was observed per each additional hour of sleep (β (95% CI) = −883.42 (−1621.78, −145.00); *p* = 0.020). This relationship was also observed for fat (β (95% CI) = −62.82 (−115.09, −10.54); *p* = 0.020) and protein (β (95% CI) = −80.11 (−152.24, −7.98); *p* = 0.031). Furthermore, a negative relation between weekday and weekend sleep efficiency and carbohydrate intake, (β (95% CI) = −0.72 (−1.41, −0.04); *p* = 0.039) and (β (95% CI) = −82.57 (−143.00, −22.14); *p* = 0.008), was identified. In the group with weekend sleep efficiency below 85% significant differences were observed for energy (β (95% CI) = −811.38 (−1509.00, 113.55); *p* = 0.023) and carbohydrate (β (95% CI) = −0.82 (−158.27, −7.12); *p* = 0.032) intake.

The relationship between actigraphic sleep duration, sleep efficiency and habitual sleep variability with dietary intake adjusted for age, sex, body fat and waist to hip ratio is presented in Table 4. For the sample as a whole and considering sleep duration as a continuous variable, a significant decrease in energy (β (95% CI) = −165.45 (−274.01, −56.88); *p* = 0.003), fat (β (95% CI) = −11.14 (−18.44, −3.84); *p* = 0.003) and protein (β (95% CI) = −13.27 (−22.52, −4.02); *p* = 0.005) intake was observed per each additional hour of sleep during the week. For the category that included all children who slept less than 9 hours a day, a significant decrease in energy (β (95% CI) = −167.08 (−290.98, −43.18); *p* = 0.008), fat (β (95% CI) = −11.11 (−19.44, −2.78); *p* = 0.0069) and protein (β (95% CI) = −13.76 (−24.30, −3.22); *p* = 0.011) intake was observed per each additional hour of sleep. The statistically significant negative relation between weekday and weekend sleep efficiency and carbohydrate intake was lost after adjustment. In the group with weekend sleep efficiency below 85%, significant differences were observed for energy (β (95% CI) = −847.43 (−1566.77, 128.09); *p* = 0.021) and carbohydrate (β (95% CI) = −83.96 (−161.76, −6.15); *p* = 0.035) intake while in the group above 85% weekend sleep efficiency, a positive relation was found with carbohydrate intake (β (95% CI) = 337.05 (3.32, 670.77); *p* = 0.048). Habitual sleep variability shows a significant positive relation with protein intake (β (95% CI) = 0.32 (0.03, 0.62); *p* = 0.031).

## 4. Discussion

The main findings of our study were that after adjustment, shorter weekday sleep duration, when studied either continuously or categorically, was related with increased energy intake coming from protein and fat, inadequate (˂85%) weekend sleep efficiency was related with increased energy and carbohydrate intake, and higher habitual sleep variability was related to higher protein intake. The relationship between lower weekday sleep efficiency and a minimal increase in carbohydrate intake was lost after adjustment. The adjustment of the multivariable linear regression analysis produces results very similar to those of the unadjusted analysis, which leads us to believe that sleep may be a variable not dependent on the confounders studied (age, sex, body fat and waist to hip ratio) when studied in relation to energy and macronutrient intake.

Prior studies have already found significant associations between sleep deprivation and higher caloric intake, both in adult [17,20,43,44,45] and pediatric populations [11,13,46]. Comparing the results of this study with adult studies, it seems that children may be more susceptible to sleep curtailment than adults [4], since adults only showed significant association when sleep duration was extremely short (<5 h) [47]. According to this study, children with <7 h of sleep would already present a significant impact on intake. Here lies the importance of developing strategies for improving sleep behaviors as the countries of southern Europe are characterized by a much shorter sleep duration than the rest of Europe [48]. In addition, a stronger association than seen in previous research [21] was observed for food intake and weekday sleep duration compared with weekend nights, suggesting that difference in behavioral patterns and daily schedule or timing between schooldays and weekends plays an important role that requires further investigation. 

With the implementation of actigraphy in research, sleep parameters relating to quantity and quality are the norm in the field, with important implications for clinical practice [49]. In this study, the low sleep efficiency compared to published sleep guidelines is striking [36,50]. This may be due to the restlessness in the sleep of children which makes actigraphy measurements less reliable than in adult studies and appropriate algorithms should be developed to improve the studies [51]. Nevertheless, actigraphy allows us to have a representative and trustworthy vision of the quality and quantity of the sleep with a non-invasive method. In this research, a negative relationship between weekday sleep efficiency and caloric intake was not found as in previous reports [45,47,52]. However, it must be noted that in this study, a significant negative correlation between weekday sleep efficiency and carbohydrate intake was found; however, the change in carbohydrate intake was minimal, not statistically significant and disappeared after adjustment, probably due to the limitations of our sample size. One possible cause of this association could be that inefficient sleep patterns on school days lead to daytime fatigue and behavioral changes [53] that induce increased intake and snacks [54]. In this study, a decreased weekend sleep efficiency was associated with an increased intake of carbohydrates, while those that did not meet the established threshold for adequate efficiency of 85% [36] also had a higher energy intake, as reported in other previous studies [45,47,52]. This relationship between worse sleep efficiency and higher caloric and carbohydrate intakes may be more marked during the weekend given that food intake during weekends is normally higher in soft drinks, other sugary drinks and fats, and lower in whole foods, leading to higher energy and carbohydrate intakes during weekends [55,56,57,58]. 

The relationship between habitual sleep variability and food intake was also investigated and a significant positive relation was found with protein intake. Other studies have found high habitual sleep variability associated with a poor obesity-promoting diet [13], perhaps because those studies studied different age groups or calculated the variable in different ways. Timing and fragmentation of sleep were not evaluated in this study and neither was time of meal consumption, since it was not required to specify the time of meals in the food diary, but recent reports have provided interesting data about the influence of late bedtime and sleep fragmentation on caloric intake [11,47,52]. Circadian timing has been analyzed previously, observing that late eating contributes to maintain a phase delay, which leads to a shorter sleep duration due to delaying bedtime, which then offers more hours available for eating, establishing a vicious circle [59]. Sleep and meal timing could be different aspects of the same obesogenic conduct pattern [11]. 

Regarding the relationship between sleep and macronutrient intake, sleep deprivation was associated with an increased consumption of fat and protein in children who slept ˂7 hours during the week. The same association was found between weekday sleep efficiency and carbohydrate intake. Macronutrient and sleep association has been widely studied, almost always observing an increase in fat intake in relation to a shorter sleep duration [17] and increased intake of snack and energy-dense food consumption high in carbohydrates [13,44,60]. However, while an increase in fat intake was observed in this study, no relevant results in relation to carbohydrates were obtained. Furthermore, the finding of the correlation between increased protein intake and reduced sleep is noteworthy, as it has not been observed in previous studies. Some reports have evidenced the association between a greater intake of proteins and obesity, basing this finding on the influence of the timing of macronutrient intake on the circadian cycle [61], particularly, eating protein 4 hours before sleep may predispose individuals to greater caloric intake [59]. Given that the present study did not assess the time of intakes, a direct relationship cannot be established. It is possible that the higher intake of protein in this study was influenced by the late timing of dinner in Spain, which is very close to bedtime and thus would favor a greater caloric intake. Further investigations would help to establish the direction of associations between sleep, meal timing and energy intake. 

Previous studies have observed a bidirectional relationship between sleep and food intake [62]. Different biological mechanisms have been proposed to explain the association between sleep and food intake. Previous research indicated that alterations in homeostatic, emotional, behavioral and environmental factors related to sleep curtailment may influence each other, explaining mechanisms linking short sleep duration and increased energy intake [63]. Curtailed sleep duration may also influence diet by simply providing more time and opportunity for eating and drinking; however, reduced sleep can also alter neuroendocrine control of appetite via changes in the levels of leptin and ghrelin [46,60,64], resulting in a hormonal state which may affect hunger and appetite predisposing overeating [14,60]. Such alterations influence response to food stimuli. When food environment is carefully controlled, sleep deprivation is associated with a lower secretion of the satiety hormone leptin and a higher secretion of hunger-stimulating hormone ghrelin, which may induce hunger feelings, and therefore, increase food intake and contribute to increased obesity [46]. Disrupted sleep increase susceptibility of brain regions associated with motivation, reward, and decision making in response to pleasurable stimuli like palatable calorie-dense and carbohydrate-rich food or snacks [5,20,65]. Furthermore, some neuroimaging studies evidenced that sleep curtailment induces an increased activity in brain reward centers in response to palatable food [54,65]. Moreover, sleep deprivation has been related to lower inhibitory impulse control, leading to increased hedonistic eating conduct [66]. Since children are still developing behavioral controls, the effect of irregular sleep pattern on emotional and impulsive eating of highly palatable food could be greater. Moreover, sleep induces daytime fatigue and stress and other emotional symptoms [67], resulting in a sensation of less energy that can increase food intake to compensate for the energy deficit caused by increased waking time [5]. For example, caffeinated drinks reduce sleep duration; however, people who are sleep deprived may consume more caffeinated drinks to feel more alert. As mentioned previously, in this study, the impact of sleep deprivation was observed from a reduction in sleep to below 7 hours in school-aged children, while adults require less than 5 h to show significant effects. However, this should be taken into consideration along with the sleep requirements for each group as children have longer sleep requirements than adults so the percentage in reduction may be similar but the overall time required for sleep remains noticeably higher in children. Given this increased vulnerability, improving sleep patterns may be especially important to optimize intervention strategies in the prevention of obesity in children, to avoid the development of impulsive or emotional conducts in front of food. 

In addition, encouraging an earlier bedtime would decrease waking time in an environment that promotes overeating. Later bedtime favors time available at night when sedentary activities are prioritized and usually accompanied by snacks or energy-dense foods that contribute to increasing energy intake [68]. The type of food consumed during or after dinner was not specifically assessed in this research, so an association cannot be established. Studying nocturnal food intakes along with the timing of them along with sleep parameters can improve the understanding of the relationship between sleep and intake [45]. 

The strengths of this study include the objective measurement of sleep parameters by actigraphy in children in this age range, as to date, most studies have only been conducted with parental or self-report surveys, a measure whose validity has not been well established, and actigraphy was usually used in children over 8 years of age. Although acute changes in sleep (1 week) were assessed, this period of time is longer than in other studies. Among the limitations is that the study sample was small and it would be necessary to enlarge it to find more significant associations. The main issue found in studies with small sample sizes is the interpretation of the results, in particular, the confidence intervals and *p*-values. Another limitation stems from the possibility of false-positive results or the overestimation of the magnitude of the association. There are also limitations associated with the statistical analysis when adjustment for confounders using methods such as multivariate linear or logistic regression is necessary. When the sample size is small, these methods can produce unreliable results. In addition, regarding dietary assessment, it should be noted that it relies on parental report and intakes during school hours cannot be objectively assessed, although the records appear to be fairly representative of real intakes. Moreover, for future research, it would be interesting to register the time of meals to have more information available to analyze the association between sleep and dietary intake. Finally, although the actigraphy variables are not as reliable as in adult research because of the restlessness in sleep in scholar age, they give a fairly accurate overview of sleep parameters in pediatric age, but it should be necessary to developed specific algorithm for children sleep assessment. 

## 5. Conclusions

In conclusion, the present study supports that decreased sleep duration is related to higher energy intake, particularly with higher protein and fat intake, lower sleep efficiency is related to higher carbohydrate intake, and higher habitual sleep variability is related to higher protein intake. These results are similar to previous findings but the mechanism underlying this link are still unclear. Further longitudinal and experimental studies are needed given the importance in identify healthy sleeping behaviors that may be integrated in nutritional intervention programs.

## Figures and Tables

**Table 1:** General characteristics.

	All (*n* = 203)	Male (*n* = 102)	Female (*n* = 101)	*P* Value
General Characteristics				
Age (years)	7.4 (1.1)	7.2 (1.1)	7.5 (1.1)	0.025
6 years old *n* (%)	59 (29.1)	36 (34.6)	23 (22.8)	0.134
7 years old *n* (%)	60 (29.6)	31 (30.4)	29 (28.7)	
8 years old *n* (%)	39 (19.2)	16 (15.7)	23 (22.8)	
9 years old *n* (%)	46 (22.7)	19 (18.6)	27 (26.7)	
BMI Z-score	0.8 (1.3)	0.9 (1.5)	0.7 (1.1)	0.159
Major Obese *n* (%)	6 (3.0)	4 (3.9)	2 (2.0)	0.342
Obese *n* (%)	30 (14.8)	19 (18.8)	11 (10.8)	
Overweight *n* (%)	51 (25.1)	23 (22.8)	28 (27.5)	
Normal *n* (%)	115 (56.7)	55 (54.5)	60 (58.8)	
Wasted *n* (%)	1 (0.5)	0 (0)	1 (1)	
Body fat (%)	15.5 (6.5)	13.7 (7	17.4 (5.3)	0.001
WHR (cm)	0.88 (0.05)	0.89 (0.46)	0.87 (0.05)	0.001
Actigraphic Sleep Parameters				
Sleep duration week (h and min)	7 h 44.1 min (54.6 min)	7 h 38.9 min (57.6 min)	7 h 49.1 min (51.3 min)	0.187
≥9 h *n* (%)	11 (5.4)	5 (4.9)	6 (5.9)	
<9 h *n* (%)	192 (94.6)	96 (95.0)	95 (94.1)	0.507
<7 h, *n* (%)	36 (17.7)	25 (24.8)	11 (10.8)	0.078
≥7 to <8 h *n* (%)	76 (37.4)	34 (33.7)	42 (41.2)	
≥8 to <9 h *n* (%)	80 (39.4)	37 (36.6)	43 (42.3)	
Sleep duration weekend (h and min)	7 h 44.8 min (1 h 5.8 min)	7 h 36.0 min (1 h 9.9 min)	7 h 53.6 min (1 h 0.4 min)	0.057
≥9 h *n* (%)	19 (9.4)	9 (8.9)	10 (9.8)	
<9 h *n* (%)	183 (90.1)	93 (62.1)	90 (88.2)	0.358
<7 h *n* (%)	45 (22.2)	29 (28.7)	16 (15.7)	0.065
≥7 to <8 h *n* (%)	72 (35.5)	37 (36.6)	35 (34.3)	
≥8 to <9 h *n* (%)	67 (33)	26 (25.7)	41 (40.2)	
Sleep Efficiency weekday (%)	80.5 (8.3)	78.9 (8.4)	82.1 (7.8)	0.005
≥85% *n* (%)	66 (32.5)	30 (29.7)	36 (35.3)	0.242
<85% *n* (%)	137 (67.5)	71 (70.3)	66 (64.7)	
Sleep Efficiency weekend (%)	79.4 (9.4)	78.9 (9.8)	79.9 (8.9)	0.454
≥85% *n* (%)	64 (31.5)	28 (27.7)	36 (35.3)	0.291
<85%, *n* (%)	139 (68.5)	73 (72.3)	66 (64.7)	
Habitual sleep variability (min)	55.8 (29.2)	57.4 (32.1)	54 (26.2)	0.429
Dietary intake				
Energy (kcal/day)	2396 (712)	2429 (839)	2363 (560)	0.512
6–8 years: Boys 1400–1700 kcal/day; Girls: 1300–1600 kcal/day *				
9 years: Boys 1800–2300 kcal/day; Girls: 1700–2000 kcal/day *				
Protein (g/day)	109.4 (60.7)	113.2 (79.2)	105.6 (33.1)	0.377
6–8 years: 19 g/day *				
9 years: 34 g/day *				
Fat (g/day)	108.8 (48.9)	110 (58.2)	107.7 (35.1)	0.746
6–8 years: 25 g/day *				
9 years: 35 g/day *				
Carbohydrate (g/day)	234.2 (41.5)	236.9 (39.1)	231.5 (43.8)	0.357
6–9 years: 130 g/day *				

Values expressed as mean (standard deviation) or *n* (%). Abbreviations: BMI, body mass index; WHR, waist to hip ratio.* Dietary Reference Intakes (Institute of Medicine of the American Academy of Sciences).

**Table 2:** Dietary intake according to actigraphic sleep parameters.

	Energy, kcal 2396 (712)	*P* Value	Fat, g 109.4 (60.7)	*P* Value	Protein, g 108.8 (48.9)	*P* Value	Carbohydrate, g 234.2 (41.5)	*P* Value
Sleep Duration								
Weekday Continuous	2397.7 (714.2)	0.002	109.0 (48.0)	0.002	109.5 (60.9)	0.003	234.2 (41.6)	0.329
Weekday Categorical								
≥9 h	2136.6 (177.1)		90.7 (10.9)		90.4 (21.1)		230.5 (34.4)	
<9 h	2412.6 (730.5)	0.005	110.1 (49.1)	0.006	110.6 (62.2)	0.007	234.4 (42.0)	0.351
<7 h	2619.1 (1328.3)	0.152	122.8 (94.1)	0.215	127.2 (128.4)	0.186	241.0 (37.3)	0.754
≥7 to <8 h	2371.8 (399.3)		107.8 (23.3)		107.3 (25.1)		233.2 (39.5)	
≥8 to <9 h	2358.4 (577.1)		106.5 (36.2)		106.2 (36.0)		232.6 (46.4)	
Weekday Continuous	2397.7 (714.2)	0.503	109.0 (48.0)	0.719	109.5 (60.9)	0.506	234.2 (41.6)	0.436
Weekend Categorical								
≥9 h	2395.1 (714.1)		109.0 (48.0)		109.5 (60.9)		232.4 (41.0)	
<9 h	2404.2 (746.7)	0.655	109.7 (50.2)	0.933	110.1 (63.7)	0.613	109.7 (50.2)	0.933
<7 h	2642.9 (1180.8)	0.940	126.3 (82.8)	0.809	123.9 (115.2)	0.785	242.6 (38.9)	0.766
≥7 to <8 h	2257.8 (422.4)		100.1 (23.9)		98.1 (22.6)		230.7 (43.5)	
≥8 to <9 h	2384.0 (563.8)		108.1 (37.3)		108.9 (35.34)		232.7 (41.7)	
Sleep Efficiency								
Weekday	2397.7 (714.2)	0.971	109.0 (48.0)	0.776	109.5 (60.9)	0.660	234.2 (41.6)	0.195
≥85%	2383.7 (1030.6)	0.842	109.7 (72.0)	0.942	114.1 (97.0)	0.817	225.8 (35.7)	0.226
<85%	2404.4 (499.8)	0.914	108.7 (30.9)	0.638	107.3 (31.5)	0.704	238.2 (43.7)	0.936
Weekend	2397.7 (714.2)	0.914	109.0 (48.0)	0.629	109.5 (60.9)	0.508	234.2 (41.6)	0.015
≥85%	2470.1 (1132.7)	0.403	115.8 (78.5)	0.517	120.1 (102.3)	0.599	227.3 (38.0)	0.052
<85%	2364.3 (396.8)	0.023	106.1 (23.4)	0.166	104.6 (23.9)	0.061	237.4 (42.9)	0.032
Habitual Sleep Variabity	2398.9 (715.8)	0.106	109.1 (48.2)	0.132	109.6 (61.0)	0.024	234.2 (41.7)	0.757

Values expressed as mean (standard deviation).

**Table 3:** Relationship between actigraphic sleep parameters and dietary intake.

	Energy, kcal		Fat, g		Protein, g		Carbohydrate, g	
	β (95% CI) *	*P* Value	β (95% CI) *	*P* Value	β (95% CI) *	*P* Value	β (95% CI) *	*P* Value
Sleep Duration (h)								
Weekday Continuous	−170.17 (−276.59, −63.76)	0.002	−11.43 (−18.59, −4.20)	0.002	−13.84 (−22.90, −4.75)	0.003	−3.14 (−9.47, 3.10)	0.329
Weekday Categorical								
≥9 h	15.79 (−459.29, 487.87)	0.941	4.75 (−24.16, 33.66)	0.713	−9.38 (−65.27, 46.51)	0.719	1.21 (−90.57, 92.80)	0.978
<9 h	−172.76 (−293.62, −51.90)	0.001	−11.53 (−19.66, −3.39)	0.006	−14.28 (−24.59, 3.97)	0.007	−3.35 (−10.43, 3.72)	0.351
<7 h	−883.42 (−1621.78, −145.00)	0.020	−62.82 (−115.09, −10.54)	0.020	−80.11 (−152.24, −7.98)	0.031	0.86 (−21.58, 23.31)	0.938
≥7 to <8 h	35.81 (−286.26, 357.88)	0.825	0.209 (−18.58, 19.00)	0.982	4.10 (−16.14, 24.35)	0.687	1.26 (−30.57, 33.11)	0.937
≥8 to <9 h	−144.70 (−639.38, 349.97)	0.562	−8.23 (−39.24, 22.76)	0.598	−2.38 (−33.33, 28.56)	0.878	−11.84 (−51.58, 27.89)	0.555
Weekend Continuous	−25.57 (−100.67, 49.52)	0.503	−0.92 (−5.98, 4.13)	0.506	−2.16 (−8.56, 4.23)	0.719	−1.73 (−6.10, 2.63)	0.436
Weekday Categorical								
≥9 h	−98.18 (−327.09, 130.72)	0.378	−4.72 (−17.97, 8.53)	0.463	−7.36 (−25.48, 10.75)	0.403	−7.11 (−27.39, 13.15)	0.469
<9 h	−25.60 (−104.37, 53.17)	0.522	−0.671 (−5.97, 4.63)	0.803	−1.92 (−8.63, 4.78)	0.573	−2.57 (−7.01, 1.95)	0.263
<7 h	−68.91 (−262.39, 124.56)	0.476	−3.27 (−16.88, 10.33)	0.630	−3.70 (−22.65, 15.25)	0.696	−5.36 (−11.57, 0.83)	0.088
≥7 to <8 h	−2.73 (−80.43, 74.97)	0.944	0.48 (−3.91, 4.88)	0.826	−1.02 (−5.18, 3.13)	0.625	−0.786 (−8.76, 7.19)	0.845
≥8 to <9 h	56.03 (−88.53, 200.61)	0.442	3.26 (−6.29, 12.82)	0.497	0.12 (−8.97, 9.23)	0.978	7.17 (−3.41, 17.75)	0.181
Sleep Efficiency (%)								
Weekday	−1.49 (−13.39–10.41)	0.805	0.13 (−0.67–0.93)	0.753	0.14 (−0.86–1.16)	0.775	−0.72 (−1.41–0.04)	0.039
≥85%	1088.11 (−9798.81–11,975.04)	0.842	−27.79 (−788.26, 732.67)	0.942	119.36 (−904.90, 1143.63)	0.817	228.19 (−144.37, 600.75)	0.226
<85%	63.47 (−1098.00, 1225.55)	0.914	17.09 (−54.61, 88.79)	0.638	−14.08 (−87.27, 59.11)	0.704	−4.51 (−105.00, 97.43)	0.936
Weekend	−57.32 (−1113.77, −999.14)	0.915	22.43 (−48.57, 93.43)	0.534	27.32 (−62.62, 117.27)	0.550	−82.57 (−143.00–22.14)	0.008
≥85%	4254.11 (−5837.14–14,345.37)	0.403	228.55 (−475.77, 929.87)	0.517	241.58 (−672.59, 1155.76)	0.599	327.31 (−3.11, 657.73)	0.052
<85%	−811.38 (−1509.00, 113.55)	0.023	−29.02 (−70.21, 12.17)	0.166	−40.48 (−82.83, 1.87)	0.061	−82.70 (−158.27, −7.12)	0.032
Habitual Sleep Variabity (min)	0.46 (−2.93, 3.84)	0.791	−0.01 (−0.24, 0.22)	0.917	0.17 (−0.11, 0.46)	0.234	−0.02 (−0.22, 0.18)	0.917

* Association coefficients are shown as β (95% CIs). β represents the change in energy (in kcal/day) or macronutrient intake (in g/day) per each addition hour of sleep, percentage point of sleep efficiency or minute of habitual sleep variability. Abbreviations: CI, confidence interval. Actigraphic and subjective sleep durations were divided into four categories.

**Table 4:** Adjusted relationship between actigraphic sleep parameters and dietary intake.

	Energy, kcal		Fat, g		Protein, g		Carbohydrate, g	
	β (95% CI) *	*P* Value	β (95% CI) *	*P* Value	β (95% CI) *	*P* value	β (95% CI) *	*P* Value
Sleep Duration (h)								
Weekday Continuous	−165,45 (−274.01, −56.88)	0.003	−11.14 (−18.44, −3.84)	0.003	−13.27 (−22.52, −4.02)	0.005	−3.13 (−9.57, 3.31)	0.339
Weekday Categorical								
≥9 h	214.5 (−414.25, 843.25)	0.421	7.92 (−36.59, 52.44)	0.666	3.93 (−79.18, 87.05)	0.908	32.46 (−126.32, 191.25)	0.339
<9 h	−167.08 (−290.98, −43.18)	0.008	−11.11 (−19.44, −2.78)	0.009	−13.76 (−24.30, −3.22)	0.011	−3.33 (−10.56, 3.90)	0.366
Weekend Continuous	−23.66 (−100.16, 52.84)	0.543	−0.95 (−6.09, 4.20)	0.717	−1.88 (−8.39,46.61)	0.567	−1.37 (−5.82,3.07)	0.543
Weekday Categorical								
≥9 h	−18.49 (−108.11, 71.13)	0.665	−1.91 (−7.36, 3.54)	0.465	−0.13 (−6.84, 6.58)	0.967	0.86 (−6.75, 8.46)	0.810
<9 h	−23.92 (−147.39, 99.57)	0.703	0.60 (−7.69, 8.90)	0.886	−2.37 (−12.88, 8.13)	0.656	−4.46 (−11.54, 2.61)	0.215
Sleep Efficiency (%)								
Weekday	−11.81 (−1177.82,1154.20)	0.984	10.56 (−67.83, 88.95)	0.791	23.59 (−75.42, 122.61)	0.639	−41.01 (−108.58, 26.56)	0.233
≥85%	2410.57 (−8937.42, 13,748.60)	0.672	90.52 (−697.63, 878.68)	0.819	267.11 (−797.83, 1332.05	0.618	145.94 (−223.97, 515.86)	0.433
<85%	79.20 (−1090.53, 1248.94)	0.894	18.34 (−52.79, 89.48)	0.611	−12.55 (−85.81, 60.72)	0.735	−4.47 (−106.80, 97.86)	0.931
Weekend	−433.78 (−1277.26, 409.69)	0.311	−10.66 (−62.17, 40.85)	0.683	−23.34 (−76.24, 29.57)	0.385	−52.57 (−126.09, 20.94)	0.160
≥85%	5294.27 (−4980.20, 15,568.74)	0.307	301.40 (−411.49, 1014.30)	0.401	335.35 (−593.30, 1264.01)	0.473	337.05 (3.32, 670.77)	0.048
<85%	−847.43 (−1566.77, 128.09)	0.021	−32.17 (−74.56, 10.22)	0.136	−40.56 (−83.94, 2.82)	0.067	−83.96 (−161.76,-6.15)	0.035
Habitual Sleep Variabity (min)	2.82 (−0.65, 6.30)	0.111	0.18 (−0.05, 0.41)	0.133	0.32 (0.03, 0.62)	0.031	−0.02 (−0.23, 0.18)	0.814

* Association coefficients are shown as β (95% CIs). β represents the change in energy (in kcal/day) or macronutrient intake (in g/day) per each additional hour of sleep, percentage point of sleep efficiency or minute of habitual sleep variability. Abbreviations: CI, confidence interval.
